# Effect of Health Education Based on Behavioral Change Theories on Self-Efficacy and Self-Management Behaviors in Patients with Chronic Heart Failure

**Published:** 2019-03

**Authors:** Hui YU, Panpan ZHANG, Xiao WANG, Yan WANG, Binyu ZHANG

**Affiliations:** Cardiovascular Surgery Ward, The First Affiliated Hospital of Harbin Medical University, Harbin 150081, P.R. China

**Keywords:** Behavioral change theories, Health education, Chronic heart failure, Self-efficacy

## Abstract

**Background:**

We aimed to explore the effect of health education and nursing intervention model based on behavioral change theories on self-efficacy and self-management behaviors in patients with chronic heart failure.

**Methods:**

Eighty-six patients with chronic heart failure treated in the First Hospital Affiliated to Harbin Medical University (Harbin, China) from November 2016 to January 2018 were enrolled in this study. The patients were evenly divided into control group and observation group (43 patients in each group), according to the random number table method. Patients in the control group received a routine health education intervention. Patients in the observation group received health education based on behavioral change theories in addition to the routine nursing intervention. The health education included mastering of the disease-related theoretical knowledge, daily self-management and exercising guidance. After 8 weeks of intervention, the self-efficacy level, self-management ability and quality of life were assessed and compared between the two groups.

**Results:**

The self-management behavior score in the observation group was higher than those in the control group (*P*=0.002). The chronic disease general self-efficacy (GSE) score in the observation group was higher than those in the control group (*P*=0.002). The quality of life (MHL) score was in the observation group was higher than those in the control group. The patient satisfaction score in the observation group was higher than those in the control group (*P*=0.021).

**Conclusion:**

Health education based on behavioral change theories can relieve symptoms of chronic heart failure, and improve patients’ self-efficacy level, self-management ability and patient satisfaction.

## Introduction

Chronic heart failure refers to a persistent state of heart failure. It is the advanced stage in heart disease progression where the heart cannot pump a sufficient amount of blood. Patients with chronic heart failure have poor prognosis, and the mortality rate is high (1). During illness, patients will be hospitalized repeatedly. Patients could lose confidence in their future due to psychological distress and physical suffering, easily leading to depression and anxiety. In the meantime, the disease results in enormous emotional and economic burden on the patients’ families (2).

Related studies (3–5) have shown that the process for recovering from chronic heart failure is slow and thus takes a long period of time. Well-managed exercise rehabilitation can improve vasodilatory function and exercise tolerance as well in patients with chronic heart failure, thereby lightening the cardiac load. It is beneficial to the recovery of cardiac function and the improvement of heart failure symptoms. Patients with chronic heart failure often require repeated hospitalization, which poses a great challenge to the patients’ physical and psychological health. Therefore, implementing health education to improve patients’ self-management abilities plays a positive role in improving the prognosis.

Health education based on behavioral change theories aims to change individual behaviors by formulating behavioral change strategies for patients. It has been widely used in clinic to improve patients’ self-management abilities, and the effect is good. The five stages of the behavioral change model are pre-contemplation, contemplation, preparation for action, action, and maintenance (3,4). Patients have different needs and motivations at different stages of the rehabilitation period. Nurses can provide scientific guidance on patients’ calorie intake and exercise based on their needs and motivations at different stages of the behavioral change model. This will help improve the patients’ random behaviors and promotes recovery (5,6).

In this work, a health education nursing program was established based on behavioral change theories. The goal was to explore the effect of health education based on behavioral change theories on self-efficacy level, self-management behaviors and quality of life in patients with chronic heart failure.

## Methods

### Subjects

Eighty-six patients with chronic heart failure who were treated in The First Hospital Affiliated to Harbin Medical University (Harbin, China) from November 2016 to January 2018 were recruited as subjects. There were 47 males and 39 females aged 31–69 years. The average age was 38.3±17.7 years. In terms of cardiac functional classification, 40 patients were assigned to class II, 31 patients were assigned to class III, and 15 patients were assigned to class IV.

The patients were evenly divided into two groups, i.e. control group and observation group (43 patients in each group), according to the random number table method. There were 20 males and 23 females in the control group. They were aged 31–65 years and had an average age of 45.1±17.4 years. There were 22 males and 21 females in the observation group. They were aged 35–69 years and had an average age of 51.5±18.6 years. There were no statistically significant differences in age, gender, and cardiac functional classification between the two groups.

Patients who met following criteria were eligible for the study: 1) patients who participated in the study signed the informed consent; 2) patients who met the diagnostic criteria for chronic heart failure; and 3) patients who were able to communicate in language and had no mental disorders and cognitive impairment. Patients who met following criteria were excluded from the study: 1) patients who had severe allergies; and 2) patients who had cognitive and communication impairments.

This study was approved by the Ethical Committee of the First Hospital Affiliated to Harbin Medical University (Harbin, China)

### Routine health education intervention for patients in the control group

Upon admission, the patients and their families were informed of the following contents. The patient’s medical condition, medical staff in charge and the ward environment were introduced. Introduction of the patient’s medical condition was focused on explaining the cause of chronic heart failure according to the doctor’s advice. Patients were also provided with information including current advancements of surgical treatment, potential postoperative discomforts and mitigation approaches, routine care procedures, drug doses, and examination process. During the treatment, the patient was informed in detail about the prohibited foods at this time. The patient was also exhorted to take medication on time according to the doctor’s advice. Various routine indicators were obtained on time by checking the patient regularly, and the examination results were shared with the patient. The patient was encouraged to perform appropriate physical exercise to relieve stress and tension.

### Health education based on behavioral change theories for patients in the observation group in addition to routine health education intervention

A nursing team was formed to implement health education nursing intervention based on behavioral change theories. The team consisted of 5 people, including 1 head nurse and 4 nursing staff who had experience in health education for more than 2 years. Team members were required to be good at communication and have a strong interest in health education research.

Doctors in our department were responsible for training the team members. The team should know the patients’ needs and motivations at different stages of the behavioral change model. A health education protocol based on behavioral change theories was finalized after thorough discussion. In terms of exercise therapy, the head nurse developed an exercise program suitable for the patient according to the patient’s physical condition, interests, hobbies, and living environment provided by the nurse in charge. The amount of exercise was also set for individual patient. The patient’s implementation of the exercise program was required to be overseen by the patient’s family. A representative from the nursing team followed up weekly to record the progress and implementation of the rehabilitation plan.

Implementation of the self-management program (7) consisted of five stages. In the pre-contemplation stage (week 1), following action items should be accomplished. In day 1, face-to-face communication was conducted with the patient and the patient’s family, aimed at building a trust relationship between the nursing team and the patient. The patient was encouraged to talk freely about his story, through which the nursing team could find the problems the patient may have. By sharing thoughts, the patient had a better understanding of his condition. After patient education about the basics of chronic heart failure and complications, the patient was also introduced to self-management strategies, including general remarks on self-management and therapeutic exercises.

In the contemplation stage (week 2), following action items should be accomplished, i.e. sharing successful self-management experience of other patients with heart failure in easy-to-understand words and discussing current risk factors and unhealthy behaviors. The goal was to emphasize the importance of self-management during illness.

In the preparation stage (weeks 3 and 4), following action items should be accomplished. A representative from the nursing team followed up regularly, and gave instructions to the patients and their families regarding various self-management techniques and dos and don’ts for exercise. The patients were organized to practice aerobic exercises such as jogging and using community recreation facilities. The nursing team developed personalized exercise capacity including intensity and duration for individual patient according to the patient’s physical condition. The patients always had information supports such as instructions of some simple exercises suitable for patients with chronic heart failure.

In the action stage (weeks 5 and 6), following action items should be accomplished. After getting up in the morning, the patients opened windows to promote natural ventilation, and performed a meditation exercise for 30 minutes. The patients learned ways to relax and maintain a positive emotional state. The patients were advised to avoid physical labor and work that required their full attention. The patients stuck to a light diet and avoided foods high in salt, protein and fat. The patients refrained from using tobacco, alcohol and caffeine, and in the meantime avoided drinking large amount of water. The patients were encouraged to follow their daily schedule to take medication, diet, do housework and exercise on time. Their daily routines needed to be recorded in time. If anything was missed, the patients should give and record an excuse truthfully. It was strictly forbidden for patients to make changes to the medication, diet, and the exercise protocol. In the meantime, other combined conditions the patients may have also needed to be cared. For example, blood pressure should be controlled if the patient had high blood pressure, and coronary artery should be controlled if the patient had coronary heart disease. The patients were required to record daily diet and exercises. The patients were assisted in behavioral change and self-management to achieve their goals. If the patient developed symptoms such as dizziness, respiratory distress, coughing up white phlegm with bubbles, etc., he/she should be sent to the hospital for treatment.

In the maintenance stage (weeks 7 and 8), following action items should be accomplished. A meeting was held every Friday afternoon. At the meeting, the patients were encouraged to further pursue their goal. Meanwhile, fellow patients, especially those patients whose condition had been improved through regular exercises and healthy lifestyle, were invited to share their own experience of daily exercises. The goal was to urge those patients who still lived an unhealthy lifestyle to make a change. In addition, the nursing team sent health education articles to patients once a week. The articles were aimed to inform patients in an illustrated way about their disease-related knowledge, the exercise goals and self-management goals at this stage, and self-management strategies, etc.

Based on patient feedback, the protocol was modified and the patient’s stage goal was updated to reflect the scientific and practical nature of the program.

### Evaluation indicators

Self-efficacy level was evaluated using the chronic disease general self-efficacy scale (GSES) developed by Stanford University, USA (8). There are 10 items in this scale including view of yourself and self-confidence when encountering difficulties. Cronbach’s α was 0.91. Each item was measured using a four-point Likert scoring scale. A higher score indicated that the patient had higher self-efficacy level.

Self-management behavior was evaluated using Ren Hongyan’s self-management behavior scale (9). The scale has 4 dimensions, i.e. daily life management, exercise management, treatment compliance management, and symptom management, including 16 items. Cronbach’s α was 0.89. Each item was measured using a four-point Likert scoring scale. A higher score indicated that the patient had stronger self-management ability.

Quality of life was assessed using the quality of life scale (MHL score) for heart failure developed by University of Minnesota (10). The scale has four dimensions, i.e. social functioning, physical roles, improvement of disease state, and emotional roles. Cronbach’s α was 0.95. The 18 items in the four dimensions were measured using a four-point Likert scoring scale. A higher score indicated that the patient had better quality of life.

Patient satisfaction was evaluated using the self-made patient satisfaction questionnaire. The questionnaire was mainly used to determine the overall satisfaction of patients with a couple of aspects such as patients’ knowledge level of chronic heart failure, health education, effective communication level of nursing team, and nursing attitude. The full score of the questionnaire was 100 points. A score of ≤60 indicated strong patient dissatisfaction. A score of 61–75 indicated patient dissatisfaction. A score of 76–85 indicated patient satisfaction, and a score of 86–100 indicated strong patient satisfaction. The validity of the questionnaire was 0.91. Patient satisfaction was calculated using the formula: satisfaction = (number of satisfaction + number of strong satisfaction) / total number of patients in the group. A total of 86 patients were followed up in this study. Of whom, 5 patients were lost to follow-up, including 2 in the observation group and 3 in the control group.

### Statistical methods

Statistical analysis was performed using the SPSS 19.0 (Chicago, IL, USA) software. Measurement data were expressed as (x ± s). The independent samples *t* test was used to compare count data. A difference was statistically significant if *P*<0.05.

## Results

### Comparison of self-management ability between the two groups

The self-management ability of patients in the observation group who received health education based on behavioral change theories was much stronger than that in the control group. The difference was statistically significant (*P*=0.028, 0.003, 0.004, 0.031, 0.002) (Table 1).

**Table 1:** Comparison of self-management ability between the two groups (x±s, point)

***Variable***	***Control group***	***Observation group***	***t***	***P***
Number of complete follow-ups	41	40		
Daily life management	15.97±1.24	17.43±2.41	2.132	0.028
Exercise management	9.94±1.08	15.13±1.82	4.598	0.003
Treatment compliance management	12.21±2.16	16.53±2.85	3.152	0.004
Symptom management	7.94±1.61	11.16±1.49	2.083	0.031
Total score	49.86±2.97	60.68±3.53	4.843	0.002

### Comparison of self-efficacy level between the two groups

The self-efficacy level of patients in the observation group who received health education based on behavioral change theories was significantly higher than that in the control group. The difference was statistically significant (*P*=0.002) (Table 2).

**Table 2:** Comparison of self-efficacy level between the two groups (x±s, point)

***Variable***	***Control group***	***Observation group***	***t***	***P***
Number of complete follow-ups	41	40		
GSES score	22.26±3.81	39.17±3.67	4.615	0.002

### Comparison of quality of life scores (MHL scores) between the two groups

The quality of life score of patients in the observation group who received health education based on behavioral change theories was significantly higher than that in the control group. The difference was statistically significant (*P*=0.039, 0.011, 0.005, 0.003, 0.000) (Table 3).

**Table 3:** Comparison of quality of life scores (MHL scores) between the two groups (x±s, point)

***Variable***	***Control group***	***Observation group***	***t***	***P***
Number of complete follow-ups	41	40		
Social functioning	12.31±0.52	17.23±0.84	1.996	0.039
Physical roles	12.37±1.43	15.53±1.94	2.751	0.011
Improvement of disease state	13.79±1.64	19.47±1.84	3.011	0.005
Emotional roles	15.15±1.24	20.28±1.26	4.286	0.003
Total score	54.27±4.06	70.39±3.98	6.952	0.000

### Comparison of patient satisfaction between the two groups

The patient satisfaction in the observation group who received health education based on behavioral change theories was significantly higher than that in the control group. The difference was statistically significant (*P*=0.021) (Table 4).

**Table 4:** Comparison of patient satisfaction between the two groups (n (%)

***Variable***	***Control group***	***Observation group***	***X^2^***	***P***
Number of complete follow-ups	41	40		
Strong satisfaction	14 (32.56)	19 (44.19)		
Satisfaction	8 (18.60)	13 (30.23)		
Dissatisfaction	17 (39.53)	8 (18.60)		
Strong dissatisfaction	4 (9.30)	3 (6.98)		
Degree of satisfaction	51.16%	74.41%	5.132	0.021

## Discussion

In the past 20 years, China has been committed to implementation of health education and health promotion programs, and has achieved great positive results in public health. As medical technology and rehabilitation medicine have advanced considerably in China, survival rate of patients with chronic diseases has been greatly improved (11). Chronic heart failure is a condition when cardiovascular disease progresses to its end stage. Due to lack of regular and healthy lifestyle, patients suffer from irregular attacks of this disease in a long period of time, which seriously affects their quality of life and puts a huge psychological and economic burden to the patients and their families as well. Therefore, in addition to clinical medication and surgical treatment, patients with chronic heart failure need to have strong self-management ability and do exercises in order to maintain a good quality of life (12).

However, traditional health education is only conveyed to the patients through simple posters and words. Because there is no clear stage guidance, the patients don’t often know what to do to improve their behavior at each stage. The goal of treatment for chronic heart failure is not only to improve symptoms, but more importantly, also to reduce mortality rate and rehospitalization rate and improve quality of life through standardized medications combined with self-management behaviors such as diet, exercise, and emotional management. Behavioral change theories aim to improve an individual’s self-management ability through continuous and dynamic processes, and to improve prognosis of patients with chronic heart failure through joint efforts of both medical staff and patients (13). Health education based on behavioral change theories, which is a new model of health education for chronic diseases, is in line with the trend of modern society development. As a service product, it can enhance the interaction between medical staff and patients, and has the functions and features of automatic feedback, openness and equality. It is a good way to better popularize postoperative rehabilitation knowledge to the patients.

Health education based on behavioral change theories consists of five stages of patient behavior change. In the pre-contemplation stage, trust is built with the patients by face-to-face communication, follow up, and sharing health education information. In the contemplation stage, the patients are encouraged to build their confidence to implement self-management. In the preparation stage, various self-management skills and exercise guidance for patients with chronic heart failure are shared to prepare the patients ready for behavior change. In the action stage, the patients are guided to make changes of their unhealthy behavior and improve themselves continuously. In the maintenance stage, positive cases are continuously conveyed to motivate the patients to maintain their good behavior and healthy lifestyles (14, 15). In different stage, the patients have different goals and needs. Thereby caregivers should deliver appropriate health education contents to them in that stage providing scientific guidance and strengthening their self-management awareness. Patients’ families are also encouraged to reward the patients after each exercise. Patients’ self-confidence should be strengthened all the time. Patients are encouraged to regard exercise as a process of enjoyment. Patients are expected to reshape their concept of health and build a good physical attribute, thereby enhancing their physical strength, improving self-management ability and self-efficacy, and eventually improving heart failure symptoms.

As the results showed, the self-efficacy level of patients in the observation group was significantly higher than that in the control group, and the difference was statistically significant (*P*<0.05). The patients communicated with their fellow patients during the gatherings, which might help improve the patients’ self-confidence. By exchanging experience between patients, the patients became more confident in achieving the goals of each stage. Thereby their self-efficacy level was improved. As the results showed, the quality of life score of patients in the observation group was significantly higher than that in the control group, and the difference was statistically significant (*P*<0.05). The nursing team received support from the patients’ families in guiding the patients to learn self-management behavior. Proper exercises improved the patients’ physical fitness. Regular lifestyle and staged behavioral change promoted improvement of the patients’ disease. Communication with fellow patients and supports from patients’ families and the nursing team continually stabilized the patients’ mood and improved their social functioning. During the process, the patients maintained a positive and optimistic attitude to their disease. Overall, these practices improved patients’ satisfaction and their quality of life.

## Conclusion

In summary, health education and nursing intervention model based on behavioral change theories can relieve symptoms of chronic heart failure, and improve patients’ self-efficacy level, self-management ability and patient satisfaction. In addition, it can effectively increase patients’ quality of life as well.

## Ethical considerations

Ethical issues (Including plagiarism, informed consent, misconduct, data fabrication and/or falsification, double publication and/or submission, redundancy, etc.) have been completely observed by the authors.
